# The effect of an educational program for pregnant women to prevent allergic diseases in infants: study protocol for a randomized controlled trial

**DOI:** 10.1186/s13063-019-3797-2

**Published:** 2019-12-21

**Authors:** Rie Nakamura, Nao Ishiguro, Eiji Naru, Yukiko Ishitsuka, Masato Nakade, Yoko Nezu

**Affiliations:** 1 KOSÉ Corporation Research Laboratories, 48-18 Sakae-cho, Kita-ku, Tokyo, 114-0005 Japan; 2 Chiba Aiyu-kai Kinen hospital, 1-1 Niregasaki, Nagareyama-shi, Chiba, 270-0161 Japan

**Keywords:** Allergies, Infant allergy, Prenatal, Pregnancy education, Allergic march, Educational intervention, Food allergy

## Abstract

**Background:**

Allergic diseases in infants have dramatically increased in developed countries during the past few decades. To date, extensive research has been done on risk factors for allergies in infancy, and preventive measures against them. However, the effect of the primary approach to preventing infantile allergy is still limited.

The aim of this trial is to evaluate whether prenatal education interventions, including the latest public research results on allergic diseases, prevent the onset of infant allergies.

**Methods/design:**

We designed a randomized controlled, two-arm (standard prenatal education vs our education), parallel-group, assessor-blind trial. A sample of 120 pregnant women will be recruited at Chiba Aiyu-kai Kinen Hospital and allocation is by computer-generated randomization.

Pregnant women in the intervention arm participate in the childbirth education program established by the specialist and a pediatric allergy educator. The program was developed based on evidences supporting interventions on primary prevention, which are suggested to be beneficial to infantile allergies in recent studies.

The primary objective of the study is to determine whether it is possible to establish effective behaviors for allergy prevention in early infancy in the children of pregnant women who participate in an educational program developed by pediatric allergy specialists. Four months after birth, their behaviors will be compared against those of pregnant women who did not participate in the program.

**Discussion:**

Allergies are common in many individuals worldwide, and can be present from babyhood through the person’s lifetime. One of the strong points of this study is that it should provide pregnant women with accumulated information on preventive knowledge against allergy, that can be effective in some cases, and that women can apply a combination of these behaviors before and after pregnancy. The results of our program will be publicized to help change the behaviors of mothers, and, if the program is effective, for preventing allergies in infants, it will be disclosed worldwide as a new preventive strategy for allergy in infants.

**Trial registration:**

UMIN-CTR, ID: UMIN000034730 Retrospectively registered on 1 December 2018.

## Introduction

In Japan, as in most developed countries, the prevalence of allergy, including atopic dermatitis (AD), food allergy (FA), asthma, and allergic rhinitis, among infants has increased in recent years and is a serious health problem [1]. The “allergic march,” which was first proposed by Minoru Baba, and refers to the chain reaction of allergies in people predisposed to atopic dermatitis [2, 3], has attracted a great deal of attention in recent years, and it has been suggested that preventative measures during the neonatal and early infancy period could be effective against this condition.

To date, extensive research has been conducted on the risk factors for, and preventative measures against, allergy during infancy. In addition, evidence supporting the efficacy of several of these preventative measures has accumulated. For example, in 2008, Lack hypothesized that sensitization to allergens occurs through environmental exposure to allergens through the skin and that consumption of food allergens induces oral tolerance (dual-allergen-exposure hypothesis [4, 5]), and proposed a new allergy-prevention strategy based on the maintenance of skin-barrier function. To verify the dual-allergen-exposure hypothesis, randomized clinical trials (RCTs) of the effects of a moisturizer were conducted, and its efficacy in AD patients was demonstrated [6–8]. Lowe et al. (2016) [9] performed a meta-analysis and showed that a moisturizer can statistically significantly prevent infantile AD when used in the neonatal period, and that the onset of AD is associated with sensitization to egg-white antigen. On the other hand, this analysis suggested that the efficacy of moisturizer alone used from the neonatal period is poor because it cannot prevent sensitization to food allergens.

In addition to the dual-allergen-exposure hypothesis, some studies suggest that maternal intestinal bacterial flora is associated with neonatal allergy [10, 11]. Currently, probiotics [12], prebiotics [13], and synbiotics [14] are effective for the treatment of abnormal intestinal bacterial flora. Some RCTs have examined the effects of probiotic intake by pregnant and lactating women on preventing the development of allergies in infants [15–19]. Zuccotti et al. (2015) [20, 21] performed a meta-analysis on these RCTs, and suggested that probiotic intake in pregnant and lactating women can reduce the incidence of rashes in neonates. However, probiotics did not reduce the prevalence of asthma or allergic rhinitis. A different meta-analysis of RCTs assessing the effects of prebiotics [22] and synbiotics [23] showed that although these organisms prevent the development of a rash, they have no protective effects against allergic sensitization in neonates. Additional studies are needed to assess the differences in intestinal bacterial flora between various races or regions.

Some observational studies identified many risk factors and preventive measures for allergy in infants. An observational study that examined the association between living conditions and infantile asthma [24, 25] reported that the combination of preventive measures to avoid allergens, such as tick and mold, can reduce the risk of allergy. Second-hand tobacco smoke from parents or other family members living together increases the risk of bronchial asthma in infants [26–28]. The association between living conditions and the risk of allergy in infants has been researched extensively. Many observational studies examined the correlation between food intake by pregnant women or lactating mothers and the development of allergies in infants. Specifically, the intake of vitamin D (VD) [29–31] and n-3 polyunsaturated fatty acids [32–34] during pregnancy reduces the risk of allergy in infants. However, there are no recent meta-analyses [35–40] or RCTs [41–46] demonstrating the efficacy of these nutrients against allergies.

The preventive measures reported to date have shown limited effects, and a clear and effective method to prevent allergies in infants remains to be identified. Because multiple risk factors are associated with allergies in infants, the preventive measures identified to date are not effective.

We hypothesized that providing pregnant women with knowledge regarding the latest research findings on allergy prevention in prenatal and postnatal period may increase awareness and knowledge and, thereby lead to changes in behavior to prevent allergy in infants. The aim of this trial is, therefore, to evaluate whether prenatal education interventions, including the latest public research results on allergic diseases, prevent the onset of infant allergies.

This protocol has been written in accordance with the Standard Protocol Items: Recommendations for Interventional Trials (SPIRIT) guideline (Additional file 1). This report is based on protocol version 9, approved 1 March 2019, by Chiba Aiyu-kai Kinen Hospital Ethics Committee.

## Methods/design

### Objectives

#### Primary objective

The primary objective of the study is to determine whether it is possible to establish effective behaviors for allergy prevention during early infancy in pregnant women who participate in an educational program developed by pediatric allergy specialists. Four months after birth, their behaviors will be compared against those of pregnant women who did not participate in the program.

#### Secondary objectives

The secondary objectives are as follows:

- To determine whether the use of a moisturizer can reduce the onset of AD and eczema
- To determine whether the onset of other atopic disorders can be prevented by the use of a moisturizer
- To examine the association between the onset of AD and food allergy sensitization
- To establish the safety of the recommended moisturizer in neonates
- To investigate the presence of allergies, skin condition, serum immunoglobulin E (IgE), and thymus and activation-regulated chemokine (TARC) levels in neonates

### Trial design

This study is an assessor-blinded, randomized controlled, parallel-group comparison study (comparing our educational program group with an established childbirth education group) conducted in Chiba Aiyu-kai Kinen Hospital (hereafter referred to as “our hospital”) to examine the superiority of the educational program over established programs. Subjects are randomized in a 1:1 ratio into one of the two groups. The participants and study personnel are not blinded with respect to the assignments.

This protocol is written in accordance with the Standard Protocol Item: Recommendations for Interventional Trials (SPIRIT) guideline. Trial registration is through the University Hospital Medical Information Network – Clinical Trials Registry (UMIN-CTR) (UMIN000034730).

### Participants

#### Recruitment procedure

Participants are recruited among all pregnant women who visit our hospital for consultation regarding delivery. The investigator meets with each pregnant woman to provide oral and written information on the study and to request participation. Participants are selected according to the eligibility criteria. The pregnant women discuss participation with their respective families, and those who agree to participate in the study provide informed consent. After the collection of consent forms, the participants are randomized to one of two groups.

#### Eligibility criteria

Eligibility and exclusion criteria are as follows:

Eligibility criteria (individuals must meet two criteria):

- Those planning to give birth in our hospital
- Those planning to attend the childbirth education program provided by our hospital

Exclusion criteria (any of the following criteria):

- Those who were not planning to give birth in our hospital
- Birth of triplets or more
- Children with serious congenital malformations
- Abnormal birth including fetal distress and neonatal asphyxia
- Other pregnant women and neonates assessed as inadequate for the study by a physician

### Intervention

#### Intervention arm

Pregnant women in the intervention arm participate in the childbirth education program established by the specialist and a pediatric allergy educator (PAE). The program was developed based on evidences supporting interventions on primary prevention, which are suggested to be beneficial to infant’s allergies in recent studies. In addition, the intervention groups will receive this program in face-to-face education. In this program, we propose three types of barriers that protect infants from allergy: the intestinal mucosa, the respiratory tract mucosa, and the skin. We then recommend behaviors for mothers that improve the functions of these three types of barriers. Table 1 explains the details of the program using the Template for Intervention Description and Replication (TIDieR) [48], a template for intervention description.

**Table 1:** Details of the educational program provided to the intervention arm

Item	Description
1. Brief name	Perinatal education to prevent allergy in infants
2. Why?	Although many parents are anxious regarding allergies and skin conditions in infants [47], there are few opportunities to learn about the prevention of pediatric allergies and skin conditions. Extensive information on pediatric allergy is available based on scientific evidence, and prophylaxis in pregnant women is expected to be effective to prevent allergy in infants. If pregnant women learn how to prevent pediatric allergy based on scientific evidence and apply these principles to childcare, this can promote the establishment of a prophylactic strategy against allergy in infants
3. What materials?	Educational materials for the program were prepared using PowerPoint. A handbook with illustrations and photographs reviewed by a specialist is provided to mothers to educate them about proper skin care for infants and the optimal diet during pregnancy and *lactation*
4. What procedures?	A session planned during early and late pregnancy consists of the following: ➢ Program in Early pregnancy Title: What you can do for your baby Subtitle: Advice from pediatric allergy specialists Intestinal mucosa ✓ Effect of the maternal intestinal bacterial flora on the fetus ✓ A diet that includes nutrients (oligosaccharides, dietary fiber, and fermented food) is ideal for pregnant women to regulate for healthy intestinal bacterial flora ✓ Effect of vitamin D and a diet that includes vitamin D ✓ Effect of n-3 polyunsaturated fatty acids and a diet that includes this acid Skin ✓ Association between the skin of infants and sensitization to allergens ✓ Adequate exposure to ultraviolet light Respiratory tract mucosa ✓ Effect of smoking on pediatric bronchial asthma ➢ Program in Late pregnancy Title: What you want to know for your baby? Subtitle: Advice from specialists on pediatric allergy: “Three barriers protecting our body” Intestinal mucosa (the same content as in Program in Early pregnancy) Skin ✓ Association between the skin of infants and sensitization to allergens ✓ Differences between the skin of infants and that of adults ✓ Prevention of allergic dermatitis and proper skin care starting at the neonatal period ✓ Proper skin care for neonates (how to wash and moisturize the skin) ✓ Proper exposure to air Respiratory tract mucosa ✓ Effect of smoking on pediatric bronchial asthma ✓ Risk of exposure of infants to allergens in the living environment, in particular in bedclothes ✓ Cleaning bedclothes and the living areas of infants
5. Who provides?	Specialist or educator of pediatric allergy
6. How?	Face-to-face group learning
7. Where?	Chiba Aiyu-kai Kinen Hospital where pregnant women are planning to give birth
8. When?	Two 90-min sessions (at approximately 20 and 30 weeks of pregnancy)
9. Tailoring	Not individualized
10. Modifications	Any changes occurring during the study period need to be reported
11. How well?	Plan: To promote adherence to the recommended skin care practices among subjects participating in the educational program. A moisturizer is provided free of charge to those who want to use it
12. How well?	Practice: Strategies to promote adherence to skin care practices will be reported in a paper

### Control arm

A leaflet summarizing the content of the educational program that is provided to the intervention arm is made available to subjects in the control arm at approximately 20 and 30 weeks of pregnancy.

### Measurements/assessments

#### Primary outcomes

##### Self-evaluation of questionnaire to identify the behavioral characteristics of mothers based on the educational program at 4 months after delivery

Primary outcomes are evaluated according to a self-evaluation score at 4 months after delivery in both arms.

The score is determined using a survey sheet created for this study (Appendix) to assess the behavioral characteristics of mothers. We prepared 21 questions to assess the dietary habits of mothers, living conditions, and the skin care of children based on the ideal behavior of mothers to prevent allergy in infant as proposed by specialists. Five options (a–e) are provided for all questions: a = 1, b = 2, c = 3, d = 4, and e = 5. Higher scores indicate more appropriate behaviors of mothers to prevent pediatric allergy at each time point.

#### Secondary outcomes

We set the following seven secondary outcomes:

1. Incidence and severity of atopic dermatitis and eczema infantile

The incidence and severity of AD and eczema in infants aged 1 week, 1 month, 4 months, and 12 months are determined by a “blinded” pediatrician according to the Clinical Practice Guidelines for the Management of Atopic Dermatitis 2016 prepared by the Japanese Dermatological Association: URL:https://www.dermatol.or.jp/uploads/uploads/files/guideline/atopicdermatitis_guideline.pdf.

2. Incidence of food allergy and sensitization to egg white, milk, and ovomucoid

The incidence of food allergy in infants aged 4 and 12 months is determined by sensitization using serum antigen-specific IgE [49], medical interview, and oral food challenge or a clear history of allergy triggered by accidentally ingested foods

3. Analysis of stratum corneum hydration, transepidermal water loss (TEWL), and microscopic and digital images

Stratum corneum hydration in infants is measured three to five times on the left lower leg, forehead, and left cheek using Skicon-200EX® (IBS Japan Co., Ltd., Shizuoka, Japan). TEWL is determined two to three times on the left lower leg, forehead, and left cheek using the VapoMeter® (DelfinTechnologies, Kuopio, Finland). Enlarged images of the skin of the left lower leg, forehead, and left cheek in infants are acquired using a USB microscope M3 (Scalar Corporation, Tokyo, Japan). Images of a wide range of skin surfaces in infants are obtained using a digital camera (Canon, Tokyo, Japan). The skin is measured and photographed at 22 °C and 50% humidity.

4. TARC level

Serum TARC level is measured in infants aged 4 and 12 months to assess the severity of AD

5. Frequency of use and amount of moisturizer

The total amount of moisturizer used during the study is calculated to predict the effect of the educational program. The moisturizer is provided free of charge to those who want to use it

6. Questionnaire survey on living habits, awareness, and knowledge of mothers before and after pregnancy

A questionnaire survey on living habits, awareness, and knowledge of mothers before and after pregnancy was prepared. Subjects answer the self-evaluation questions in the survey at 30 weeks of pregnancy, and at 1, 4, and 12 months after delivery. Questions on living habits address stool frequency, use of supplements, eating habits between meals, and foods avoided or foods eaten. Regarding awareness and knowledge, the questions address what the mothers care about before and after pregnancy, how they intend to provide nourishment for the baby, and their concerns regarding childcare and the skin care of the baby.

7. Adverse events

Self-reported adverse events associated with the use of the moisturizer provided free of charge to those who want to use it during the study are recorded.

### Baseline characteristics

The baseline characteristics are evaluated at approximately 20 weeks of pregnancy. The variables to be measured include age; delivery experience; history and type of allergy of the subject, their partner, and siblings; type of pet, if any; and smoking status of the subject and her family members living together.

### Participant timeline

Table 2 shows the consent forms obtained from the subjects, the intervention, and the evaluation schedule.

**Table 2:** Consent, enrolment/allocation, and assessment of subjects (Additional file 2)

	Study period							
	Enrollment Allocation	Post allocation						
Time point	Early pregnancy	20 weeks of pregnancy	30 weeks of pregnancy	Delivery	Discharge	1 month after delivery	4 months after delivery	12 months after delivery
Enrollment/allocation:								
Inclusion	●							
Obtaining informed consent	●							
Randomization	●							
Baseline survey		●						
Interventions:								
Educational program		●	●					
Established childbirth education		●	●					
Assessments:								
Questionnaire survey on living habits, awareness, and knowledge of mothers before and after pregnancy			●			●	●	●
Survey on the behavioral characteristics of mothers						●	●	●
Blood collection from mothers	●		●					
Blood collection from infants (IgE and TARC)							●	●
Stratum corneum hydration of infants					●	●	●	
TEWL of infants					●	●	●	
Findings on the skin of infants (scoring atopic dermatitis (SCORAD)) [50]					●	●	●	
Images of the skin of infants					●	●	●	
Diagnosis of atopic dermatitis in infants							●	●
Diagnosis of food allergy in infants							●	●
Provision of moisturizer					●	●	●	
Collection of the moisturizer container						●	●	●

Legend: *IgE* immunoglobulin E, *SCORAD* scoring atopic dermatitis, *TARC* thymus and activation-regulated chemokine, TEWL transdermal water loss

### Sample size

In this study, the number of deliveries per month at the investigational site was estimated at 15–20, or more than approximately 200 childbirths per year. Moreover, with the current Japanese infant medical checkup system, it is unlikely that the mother will avoid the hospital in the first 4 months after delivery. Therefore, assuming a minimal recruitment rate of 70% and a less than 10% dropout rate, we expect a total of 120 (200 × 0.7 × 0.9 = 126) participants in our study. A questionnaire aimed at identifying the behavioral characteristics of mothers was created for the study to assess primary outcomes. Currently, there are no data showing the effectiveness of the survey sheet. Considering the inclusion of 60 subjects per group with a significance level of 0.05 and a standard deviation of 16 under the conditions described above, the expected upper limit of the standard error of the mean was approximately eight using a *t* test with a statistical power of 80%. Based on this estimate, the final target sample size was set at 120 patients. The statistical power was calculated using R ver.3.5.2 statistical software (R Foundation for Statistical Computing, Vienna, Austria).

### Randomization procedure and concealment of allocation

Participants who meet the eligibility criteria and agree to participate in the study are randomized after signing the informed consent form. These subjects are stratified according to the estimated delivery date into winter and other seasons, and randomized using the permuted-block method. A statistical analyst generates a random allocation code using R statistical software (R Foundation for Statistical Computing) to randomize the subjects at each investigational site. The study is an assessor-blinded trial, and all raters are blinded with respect to the assignments. Also only the statistical analyst is aware of the stratification block size; the remaining staff is blinded. Subjects are randomized in a 1:1 ratio into one of two groups.

### Statistical analysis

#### Full analysis set

Subjects enrolled in the study who started any of the study procedures are included in the full analysis set (FAS). However, those with a confirmed serious deviation from the protocol and those determined ineligible for the study after enrollment are excluded from the FAS.

#### Intention-to-treat analysis

In general, all statistical analyses are performed by the intention-to-treat principle.

### Primary outcomes

#### Self-evaluation of questionnaire to identify the behavioral characteristics of mothers based on the educational program at 4 months after delivery

A linear mixed-effects model is used to analyze the total score of the questionnaire at 1, 4, and 12 months after delivery as an outcome variable. A mixed-effects model for repeated measurements (MMRM) [51] is used to properly handle missing values or dropouts that could arise during the study. An unstructured covariance structure is used to assess the correlation between outcomes at these time points. Mean changes from baseline are modeled as outcome variables for the MMRM. Mean changes from baseline at each time point are analyzed individually using a regression model. If the regression parameters cannot be estimated using the unstructured model, the covariance structure is changed in the following sequence: Toeplitz matrix model, heterogeneous compound symmetry model, autoregressive model (1), compound symmetry model, and variance components model. A primary analysis is performed using estimates for the regression parameter or regression parameter test at 4 months after delivery. The significance level is set at 5% (two-sided).

### Secondary outcomes

#### Incidence and severity of atopic dermatitis and eczema infantile

The incidence of infantile AD and eczema infantile is compared between groups at all time points using Pearson’s chi-squared test. The severity of these diseases is compared between groups using descriptive statistics of scores at each time point. Subgroup analyses are performed to assess the incidence and severity of AD related to the amount and frequency of moisturizer used during the study and the skin condition of infants.

#### Incidence of food allergy and sensitization to egg white, milk, and ovomucoid

The rate of sensitization to each allergen is compared between groups with a cut-off value of the incidence of food allergy and the serum antigen-specific IgE level (equal to or more than class II) in infants at each time point using Pearson’s chi-squared test. Subgroup analysis is performed to assess the outcomes related to the amount and frequency of moisturizer used during the study and the onset of AD in infants.

#### Analysis of stratum corneum hydration, TEWL, and microscopic and digital images

Descriptive statistics of the stratum corneum hydration, TEWL in infants and rate of changes in the score at each time point are calculated and compared between groups. Subgroup analysis is performed to assess the amount and frequency of moisturizer used in infants during the study. In addition, an exploratory investigation is performed to evaluate the skin images.

#### TARC level

Descriptive statistics of the serum TARC level at each time point are calculated and compared between groups. Subgroup analysis is performed to assess the incidence of infantile AD and eczema infantile, and the amount and frequency of moisturizer used during the study.

#### Frequency of use and amount of moisturizer

Descriptive statistics of the total amount of moisturizer and rate of changes in the scores at each time point are calculated and compared between groups. An evaluation is performed considering birth month as a baseline characteristic.

#### Questionnaire survey on living habits, awareness, and knowledge of mothers before and after pregnancy

Descriptive statistics of a questionnaire survey’s scores at each time point are calculated and compared between groups. Changes in the self-evaluation at each time point and the related factors are compared between groups.

#### Interim analysis

No interim analysis will be conducted.

#### Data collection procedure

All data will be analyzed by members specifically trained in the study protocol in our hospital.

### Dissemination

#### Research ethics approval

The study will be performed according to the Declaration of Helsinki (adopted by the 18th World Medical Association General Assembly, 1964, including subsequent revisions) and the Ethical Guidelines for Medical and Health Research Involving Human Subjects (Public Notice of the Ministry of Education, Culture, Sports, Science and Technology, and Ministry of Health, Labour and Welfare No. 3 of December, 2014). This study was approved by our hospital ethics committee.

#### Protocol amendments

Any amendments to the protocol will be submitted to the Ethics Committee of Chiba Aiyu-kai Kinen Hospital for approval. Once approved, they will be reported to all the study investigators and, when necessary, to the study participants.

#### Confidentiality

All personnel involved in this study will comply with the applicable laws and regulations regarding confidentiality. Study personnel must make every effort to protect confidential information on the subjects and must not disclose the information obtained during the study to any third party without due cause. This is binding for any personnel even after they are no longer directly associated with the study. The investigator, the person in charge of the study, and study collaborators will use subject identification codes or registration numbers to enroll subjects and prepare case report forms instead of personal information that could be identified by a third party such as name, initials, address, telephone number, or medical chart number. With respect to information disclosure, the investigator or other personnel must carefully handle study data on the subjects to ensure that the data are not disclosed.

#### Research funding and conflicts of interest

The authors declare no conflicts of interest related to the plan, implementation, and reporting of the study results and interpretation of the results. The researchers independently planned, and will conduct, this study without funding or benefits from a company. Subjects do not lose any right or benefit by participating in this study.

#### Safety of subjects and compensation related to participation in the study

Subjects will need to allocate a certain amount of time to the study as follows: approximately 180 min for participation in sessions at 20 and 30 weeks of pregnancy, approximately 30 min for completing the questionnaire survey, and approximately 30 min for skin measurements at each time point. No special compensation is paid because adverse health effects are not expected, as the aim of the study is to educate subjects. However, if subjects experience any abnormal feelings during blood collection in the study, they must promptly contact the investigator to receive proper treatment.

#### Dissemination policy

The full protocol will be published in an academic journal in English. The study results will be announced to the public through presentations in academic meetings and publications in academic journals.

## Discussion

Allergy is one of the most important health issues in developed countries. Allergies are common in many individuals worldwide, and can be present from babyhood through the person’s lifetime. Many observational studies and RCTs have been performed to address the prevalence of allergies. These studies revealed that specific intervention strategies to prevent allergies are effective in some cases.

One of the strong points of this study is that it should provide pregnant women with accumulated information on preventive knowledge against allergy that can be effective in some cases, and the women can apply a combination of these behaviors before and after pregnancy. However, our trial has a few limitations. First, the trial may not provide clear data on the association between individual preventive measures and risk factors for allergy because the mothers themselves will be responsible for selecting and applying a combination of preventive measures. The second limitation is the study population because our sample is a relatively homogeneous group of pregnant women who are giving birth in a single hospital. Therefore, our findings may not be generalizable to other institutions or communities. However, at least in present-day Japan, a system for pregnant women to receive education is already in place, so we hope that all hospitals will have the opportunity to conduct our educational program.

Currently, most pregnant women in Japan are concerned about the potential development of allergies in their babies. If the results of the study indicate that the educational program has a positive effect on preventing allergies, “prenatal education to prevent allergy in infants” should be added to the existing childbirth educational program provided by medical institutions and local governments or the physician’s instructions provided during infant examinations. This may decrease the anxiety of pregnant women and prevent allergy in infants. Our educational program is based on research findings from papers published in international medical journals. Thus, this program, which is the first study to determine the effect of education on allergy prevention, is expected to be effective both within and outside the Japanese population. The results of our program will be publicized to help change the behaviors of mothers and, if the program is effective for preventing allergies in infants, it will be disclosed worldwide as a new preventive approach for allergy in infants.

## Trial status

Participant recruitment started in May 2019 and is scheduled to end in April 2020. This report is based on protocol version 9.

### Supplementary information

**Additional file 1.** Standard Protocol Items: Recommendations for Interventional Trial (SPIRIT) 2013 Checklist: recommended items to address in a clinical trial protocol and related documents.

**Additional file 2.** Consent, enrolment/allocation, and assessment of subjects according to SPRIT format.

## Data Availability

Data sharing is not applicable to this article as no datasets were generated or analyzed during the current study.

## Appendix

**Table 3:** Self-evaluation of questionnaire at 4 months after delivery

Question	Options				
How often do you vacuum the bed pad?	Never	Once a month	Once a week	2 or 3 times a week	Every day
How often do you wash the bedclothes?	Never	Once a month	Once a week	2 or 3 times a week	Every day
Are there any smokers living together? If yes, where do they smoke?	Yes Any place	Yes Under a ventilator	Yes Only outside the room	Yes Only outside the home	No one at all
Do you smoke? If yes, how often do you smoke?	Yes Frequently	Yes Often	Yes Sometimes	No But used to smoke	No Never smoked
How long are you be exposed to sunlight?	Not at all	Less than 30 min a day	30 min to 1 h a day	1 to 2 h a day	2 h or more a day
How often do you use sunscreen?	Never	Rarely	Sometimes	Everyday	2 times a day
How often do you take your baby outside?	Never	As long as possible	As short as possible	Not conscious	Short time with conscious
How often do you eat fish?	Less than once a week	1 or 2 times a week	3 or 4 times a week	5 or 6 times a week	Every day
How often do you eat eggs?	Less than once a week	1 or 2 times a week	3 or 4 times a week	5 or 6 times a week	Every day
How often do you eat meat?	Less than once a week	1 or 2 times a week	3 or 4 times a week	5 or 6 times a week	Every day
How often do you eat mushrooms?	Less than once a week	1 or 2 times a week	3 or 4 times a week	5 or 6 times a week	Every day
How often do you eat foods containing *Lactobacillus*?	Less than once a week	1 or 2 times a week	3 or 4 times a week	5 or 6 times a week	Every day
How often do you eat seaweeds?	Less than once a week	1 or 2 times a week	3 or 4 times a week	5 or 6 times a week	Every day
How often do you use soap when bathing your baby?	Less than once a week	1 or 2 times a week	3 or 4 times a week	5 or 6 times a week	Every day
How often do you wash your baby with a lathered soap?	Never	Rarely	Sometimes	Frequently	Always
How do you wash your baby’s face?	With hot water	Wear with gauze	Only cheeks or round mouth with soap	Whole face with soap but avoiding eyes	Whole face with soap
How do you wash your baby’s body?	Not at all	With hot water	Wear with gauze	Whole body with soap but avoiding eyes	Whole body with soap
How often do you use moisturizer for your baby?	Never	Rarely	Serious skin problems occur	Any skin problems occur	Every day
How much moisturizer do you use for your baby?	Not at all	Rubbing	Thinly	Thinly or plenty depend on body part	Plenty
How often do you use moisturizer for your baby?	Never	Rarely	Once a day if possible	Once a day at least	More than 2 times a day
Which part of your baby do you use moisturizer?	Not at all	Arms and legs	Only face	Only body	Face and whole body

Please select the one that best fits your current situation

## Abbreviations

AD: Atopic dermatitis
CI: Confidence interval
FA: Food allergy
PAE: Pediatric allergy educator
RCT: Randomized controlled trial
SCORAD: Scoring atopic dermatitis
TARC: Thymus and activation-regulated chemokine
UMIN-CTR: University Hospital Medical Information Network – Clinical Trials Registry
VD: Vitamin D
