# Supplementary material for: The effect of an educational program for pregnant women to prevent allergic diseases in infants: study protocol for a randomized controlled trial
Source: Trials. 2019 Dec 21;20:755. doi: 10.1186/s13063-019-3797-2 (PMC6925408; doi:10.1186/s13063-019-3797-2)
Supplement: Supplementary file 2 — Additional file 2. Consent, enrolment/allocation, and assessment of subjects according to SPRIT format. [file 13063_2019_3797_MOESM2_ESM.doc]

Additional file 2 Consent, enrolment/allocation, and assessment of subjects according to SPRIT format

|  | **STUDY PERIOD** | | | | | | | |
| --- | --- | --- | --- | --- | --- | --- | --- | --- |
|  | **Enrolment**  **Allocation** | **Post-allocation** | | | | | | |
| **TIMEPOINT**** | ***Early pregnancy*** | ***20 weeks of pregnancy*** | ***30 weeks of pregnancy*** | ***Delivery*** | ***Discharge*** | ***1 month after delivery*** | ***4 month after delivery*** | ***12 month after delivery*** |
| **ENROLMENT:** |  |  |  |  |  |  |  |  |
| **Eligibility screen** | X |  |  |  |  |  |  |  |
| **Informed consent** | X |  |  |  |  |  |  |  |
| **Allocation** | X |  |  |  |  |  |  |  |
| **INTERVENTIONS:** |  |  |  |  |  |  |  |  |
| **Educational program through face-to-face learning** |  |  |  |  |  |  |  |  |
| **Educational program through summary leaflet** |  |  |  |  |  |  | X |  |
| **ASSESSMENTS:** |  |  |  |  |  |  |  |  |
| **Questionnaire survey on living habits, awareness, and knowledge of mothers before and after pregnancy** |  |  | X |  |  |  | X | X |
| **Survey on the behavioral characteristics of mothers** |  |  | X |  |  |  |  | X |
| **Blood collection from mothers** | X |  | X |  |  |  | X | X |
| **Blood collection from infants (IgE and TARC)** |  |  |  |  |  |  | X | X |
| **Stratum corneum hydration of infants** |  |  |  |  | X | X | X |  |
| **TEWL of infants** |  |  |  |  | X | X | X |  |
| **Findings on the skin of infants (scoring atopic dermatitis [SCORAD]) ^50)^** |  |  |  |  | X | X | X |  |
| **Images of the skin of infants** |  |  |  |  | X | X | X |  |
| **Diagnosis of atopic dermatitis in infants** |  |  |  |  |  |  | X | X |
| **Diagnosis of food allergy in infants** |  |  |  |  |  |  | X | X |
| **Provision of moisturizer** |  |  |  |  | X | X | X |  |
| **Collection of the moisturizer container** |  |  |  |  |  | X | X | X |

*Recommended content can be displayed using various schematic formats. See SPIRIT 2013 Explanation and Elaboration for examples from protocols.

**List specific timepoints in this row.
